# STI in remote communities: improved and enhanced primary health care (STRIVE) study protocol: a cluster randomised controlled trial comparing ‘usual practice’ STI care to enhanced care in remote primary health care services in Australia

**DOI:** 10.1186/1471-2334-13-425

**Published:** 2013-09-09

**Authors:** James Ward, Skye McGregor, Rebecca J Guy, Alice R Rumbold, Linda Garton, Bronwyn J Silver, Debbie Taylor-Thomson, Belinda Hengel, Janet Knox, Amalie Dyda, Matthew G Law, Handan Wand, Basil Donovan, Christopher K Fairley, Steven Skov, Donna Ah Chee, John Boffa, David Glance, Robyn McDermott, Lisa Maher, John M Kaldor

**Affiliations:** 1 Baker IDI, Alice Springs, Northern Territory 0870, Australia; 2 The Kirby Institute, University of New South Wales, Sydney, NSW 2052, Australia; 3 Discipline of Obstetrics and Gynaecology, The University of Adelaide, Adelaide, SA 5005, Australia; 4 Epidemiology and Health Systems Division, Menzies School of Health Research, PO Box 41096, Casuarina, NT 0811, Australia; 5 Northern Territory Department of Health, 55 North Stuart Highway, Alice Springs 0870, Australia; 6 Apunipima Cape York Health Council, Bungalow, Queensland 4870, Australia; 7 Melbourne Sexual Health Centre, Alfred Health, Carlton, Victoria, Australia; 8 School of Population Health, University of Melbourne, Parkville, Victoria, Australia; 9 Central Australian Aboriginal Congress, Alice Springs, Northern Territory 0870, Australia; 10 University of Western Australia, Perth, Western Australia 6009, Australia; 11 Sansom Institute of Health Service, School of Health Sciences, University of South Australia City East Campus North Terrace, Adelaide, SA 5000, Australia; 12 Centre for Disease Control, Northern Territory Department of Health, Rocklands Drive, Tiwi 0811, Australia

**Keywords:** Aboriginal, Indigenous, Sexually transmitted infections, Chlamydia, Gonorrhoea, Trichomonas, Continuous quality improvement, Protocol, Prevalence, Remote

## Abstract

**Background:**

Despite two decades of interventions, rates of sexually transmissible infections (STI) in remote Australian Aboriginal communities remain unacceptably high. Routine notifications data from 2011 indicate rates of chlamydia and gonorrhoea among Aboriginal people in remote settings were 8 and 61 times higher respectively than in the non-Indigenous population.

**Methods/design:**

STRIVE is a stepped-wedge cluster randomised trial designed to compare a sexual health quality improvement program (SHQIP) to usual STI clinical care delivered in remote primary health care services. The SHQIP is a multifaceted intervention comprising annual assessments of sexual health service delivery, implementation of a sexual health action plan, six-monthly clinical service activity data reports, regular feedback meetings with a regional coordinator, training and financial incentive payments. The trial clusters comprise either a single community or several communities grouped together based on geographic proximity and cultural ties. The primary outcomes are: prevalence of chlamydia, gonorrhoea and trichomonas in Aboriginal residents aged 16–34 years, and performance in clinical management of STIs based on best practice indicators. STRIVE will be conducted over five years comprising one and a half years of trial initiation and community consultation, three years of trial conditions, and a half year of data analysis. The trial was initiated in 68 remote Aboriginal health services in the Northern Territory, Queensland and Western Australia.

**Discussion:**

STRIVE is the first cluster randomised trial in STI care in remote Aboriginal health services. The trial will provide evidence to inform future culturally appropriate STI clinical care and control strategies in communities with high STI rates.

**Trial registration:**

Australian and New Zealand Clinical Trials Registry ACTRN12610000358044

## Background

For almost two decades sexually transmissible infection (STI) diagnoses have occurred at hyperendemic rates in many remote Australian Aboriginal and Torres Strait Islander (hereafter referred to as ‘Aboriginal’) communities [1]. Data on prevalent and incident infection in these settings are derived from routine notifications reportable by public health agencies and sporadic prevalence surveys conducted in the context of screening programs [2].

In 2011 the diagnosis rates of *Chlamydia trachomatis* (chlamydia) and *Neisseria gonorrhoeae* (gonorrhoea) among Aboriginal people resident in remote communities were 8 and 61 times greater than in the non-Indigenous population [1]. A similar situation exists for *Trichomonas vaginalis* (trichomonas) in the Northern Territory, the only jurisdiction which requires notification of this infection [3]. The majority of these three STIs are notified among people aged 15–29 years [1].

While differential patterns of testing may play some role in these discrepant rates of diagnosis, a major component of the disparity [1] is almost certainly attributable to inadequate access to early detection and appropriate treatment programs offered in primary health care settings [4].

Chlamydia, gonorrhoea, and trichomonas are fully curable STIs but are often asymptomatic for long periods and can lead to serious complications if untreated [5]. For example, untreated chlamydia infection may potentially cause pelvic inflammatory disease (PID), ectopic pregnancy and tubal factor infertility among women [6,7]. In the Top End of the Northern Territory, 26% of all Aboriginal women were found to have had history of PID [8]. Chlamydia can also result in adverse pregnancy outcomes including premature labour and birth, low birth weight, intrauterine growth restrictions, postpartum endometritis [9,10] and a range of neonatal infections including infectious conjunctivitis and pneumonitis [5]. Gonorrhoea has similar sequelae to chlamydia and can also lead to disseminated infection [5]. Trichomoniasis among women is associated with premature rupture of membranes, premature delivery and low birth weight [11,12] while trichomoniasis in men is an important cause of non gonococcal urethritis and is associated with prostatitis [13,14]. All three STIs are associated with an increased risk of HIV transmission [15,16].

### STI programs in remote aboriginal communities

Despite many years of effort aimed at reducing the incidence and prevalence of STIs in remote communities, it is speculated that increased support to primary care is required to achieve best practice in the clinical management of STIs. In this setting, staff turnover is high and the burden of chronic disease, other infectious diseases, and child and maternal health needs is particularly high [17,18]. Given that resources are finite, particularly in regard to the availability of clinical personnel, several approaches have been used to support specialised areas of health service need in remote communities. One method has been to appoint specialist visiting personnel to provide service delivery in primary care settings at a regional level, focusing exclusively on the area of need [2,19,20]. These activities are recognized as having the fundamental limitation that they are unlikely to be well enough resourced to provide specialised assistance to all, or even a high percentage, of clinical episodes. In the area of sexual health, population screening has been used in some communities to improve testing coverage, but generally these programs depend on outside teams providing a service within a limited time frame, and may not always contribute to the confidence and competence of local clinical staff.

In addition to detecting and treating infections, a major goal of improving sexual health service delivery is to reduce the overall prevalence of infections. Analyses of program data to compare changes in STI prevalence over time in communities with different levels of service activity provide some insight into the effectiveness of programs [2], but their interpretation is compromised by a number of potential confounding effects. These effects include differences between communities, such as size and mobility of the population, sexual networks and the extent of treatment-seeking behaviours [2], all of which are difficult to monitor systematically. Another approach is to randomly allocate selected communities to receive a program of support over a defined time period to enhance STI diagnosis and treatment to best practice levels and to compare outcomes with control communities. This approach has been trialed in remote primary health care services addressing improvements in quality of care related to chronic disease [17,21-23].

The primary objectives of the STRIVE trial are:

1. To determine whether targeted support to health services can achieve substantive and sustained improvements in the provision of sexual health clinical services in remote Aboriginal communities; and

2. To determine whether the attainment of best practice levels in clinical activity can reduce the prevalence of curable STIs in these communities.

## Methods

### Study design

STRIVE is a community-based stepped-wedge cluster randomised controlled trial. During the first 18 months of the trial, 24 ‘trial clusters’ comprising 68 remote communities and primary care centres in Central and Northern Australia were recruited to participate in the trial, and undergo preparatory procedures. The trial clusters are the unit of randomization and comprise either a single community or several communities that share close cultural affinities and/or are geographically proximal. The clusters were determined by the research team in consultation with stakeholders and are located in remote Australia, in the Northern Territory (18 clusters), Western Australia (3 clusters) and Queensland (3 clusters).

**Figure 1:**
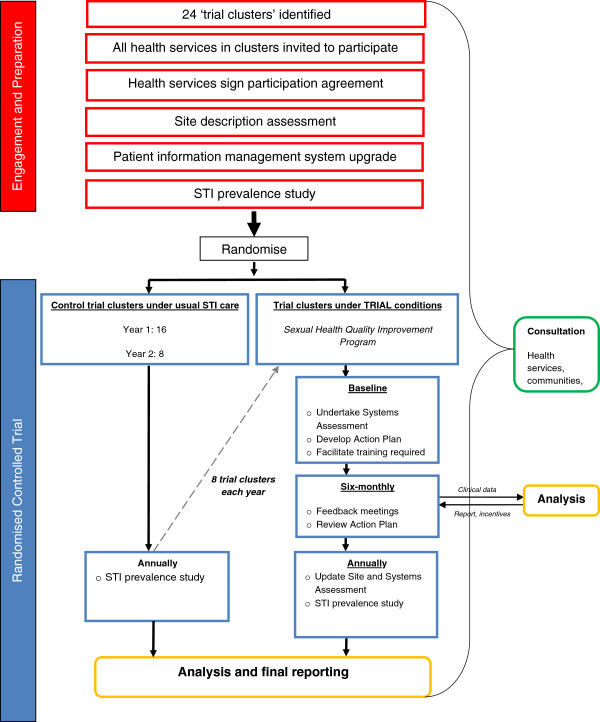
STRIVE study process.

At the start of each of the three years, one third of the clusters are randomly assigned to receive the SHQIP (see Figure 1). Clusters not randomised to receive the SHQIP continue to undertake usual STI care. By the start of year four, all clusters will be receiving the SHQIP.

The stepped-wedge design [24] ensures that all communities and their health services in the trial receive the SHQIP. Given that this design enables the intervention to be delivered to all clusters and that the intervention is likely to have a potential beneficial effect [25] it was affirmed as a preferred model with key stakeholders.

Chlamydia, gonorrhoea and trichomonas are good candidates for clinical quality improvement strategies because they are endemic in remote Aboriginal communities and can be easily diagnosed and treated. Moreover in the same communities infectious syphilis [26] and donovanosis [27] have declined significantly over the last decade as a result of sustained and specific targeted clinical programs. While STRIVE focuses on the three specified STIs, it also encourages ‘best practice’ for the detection and treatment of all STIs.

### Ethics and informed consent

During 2009–2010 the STRIVE study protocol was approved by the Central Australian Human Research Ethics Committee, the Cairns and Hinterland Human Research Ethics Committee, the Human Research Ethics Committee of the Northern Territory Department of Health and Menzies School of Health Research, the University of New South Wales Human Research Ethics Committee, the Western Australian Aboriginal Health Information Ethics Committee, and the Western Australian Country Health Service Board Research Ethics Committee.

Participating health services signed a site participation agreement prior to commencement of their involvement in STRIVE. Consultation and laboratory data are collected through routine patient management systems.

### Community engagement and collaborations

A key underlying principle of STRIVE is effective engagement at all stages with stakeholders including government, non-government organisations and the community. Extensive community engagement occurs through multiple modes including engagement with key stakeholders, an initial community engagement workshop and constant communication with participating health services.

### Identification of communities and health services and eligibility criteria

For inclusion in STRIVE, communities must be considered remote by the Australian Bureau of Statistics [28]; have a resident population of at least 100 Aboriginal people aged 16–34 years and Aboriginal people must comprise a majority of the population; have a health service willing to participate based on the trial protocol including an ability to provide access to de-identified clinical data throughout the trial. Study personnel determined eligibility of communities and health services to participate. All eligible communities and health services within each trial cluster were invited to participate. Conversely a community is not eligible if there are multiple health services in that community.

### Randomisation

Towards the end of the preparatory period, eight trial clusters (designated “first year trial clusters”) were randomly selected to receive the SHQIP in the second year. Towards the end of the second year of STRIVE, a further eight trial clusters were randomly allocated to the SHQIP in the third year and fourth years.

Randomisation occurs after participation agreements have been signed and the baseline STI prevalence survey has been conducted. Trial clusters are randomised to the SHQIP or usual care using a minimisation approach that aims to balance population testing coverage rates, cluster population size and geographic region across the baseline levels in each trial cluster. The statistician, who was blinded to the study sites (regions), prepared the randomisation list for each geographical area. The protocol was implemented in four regions of Australia in mid 2010.

### Baseline activities conducted with participating health services

At enrolment, all participating primary care services undertake three preparatory steps, supported by one of the STRIVE study coordinators, each of whom works on a regional basis. Step 1 is a site assessment to provide information on the health service context (staffing, location, pathology provider, patient information management system) and both current and past sexual health activities; Step 2 involves the development and installation of an upgraded STI template within the service’s patient information management system. Step 3 involves provision by the coordinator of training and support to conduct a STI prevalence survey with the service.

### The sexual health quality improvement program (SHQIP)

The SHQIP is the intervention that takes place at each health service, beginning in the year that the cluster to which it belongs is selected by the randomisation process. It is a multifaceted program, that involves an annual assessment covering six key areas considered as STI best practice (a systems assessment) followed by the development of an action plan, both undertaken with the support of a STRIVE coordinator. At six-monthly intervals, the service (both management and clinical staff) receives quantitative reports from patient information systems and laboratory data on clinical activities based on a set of quality indicators (see Table 1) and participates in collaborative feedback meetings with STRIVE coordinators to review and address needs in sexual health clinical service delivery. The coordinators also identify and address requirements for training in sexual health service delivery. Services assigned to the SHQIP receive a one-off payment of $2,000 to support health promotion activities aimed at increasing clinical visits for STI testing by young people, and at six monthly intervals they receive payments calculated on the basis of their performance against the best practice indicators (described below).

**Table 1:** STI best practice indicators

**STI best practice indicators**	**Target**
Proportion of Aboriginal patients aged 16–34 years old who have an annual test for chlamydia, gonorrhoea and trichomonas	80%
Proportion of health service patients presenting with STI symptoms who receive immediate treatment	95%
Proportion of people diagnosed by laboratory test with chlamydia, gonorrhoea or trichomonas who are treated within seven days of the test result being received from the laboratory	80%
Proportion of patients found by laboratory test to have chlamydia, gonorrhoea or trichomonas who have a test for re-infection at between two to four months following treatment	80%
Proportion of named sexual contacts, of people found to have chlamydia, gonorrhoea or trichomonas, tested and treated	50%

#### Systems assessment

STRIVE coordinators work collaboratively with health service staff to assess the current status of sexual health service delivery using a self-assessment tool based on the methods of the Audit and Best Chronic Disease Extension program [29], The tool enables any gaps in STI care and management to be identified, and explores ways to improve sexual health clinical services in relation to the diagnosis and management of STIs. The systems assessment covers six areas; including health hardware, clinical services, information systems, health promotion, organisational commitment, surveillance and evaluation. Staff score their health service on an 11-point scale in each area, with an aim of improving areas at subsequent annual assessments. The areas cover the fundamental components of a comprehensive sexual health program identified for the remote primary health care setting [30].

### Best practice STI indicators

The indicators established for STRIVE (Table 1) are based on current guidelines for best practice in STI care and management [31-33]. According to these guidelines, young people at risk of STIs should have a test for chlamydia, gonorrhoea and trichomonas every year; syndromically treated at consultation, and as soon as possible following a laboratory diagnosis. Those treated for an infection(s) should be asked to refer or name sexual contacts to clinic staff, and return for a test for re-infection for gonorrhoea and chlamydia at three months. The indicators in Table 1 are discussed with health service staff at the first systems assessment meeting, and form the basis for subsequent reports on performance. Each indicator is calculated for six month periods, apart from the annual screen. Indicator calculations are based on Aboriginal residents as determined by the health service.

#### Sexual health action plan

The action plan is based on the outcome of the annual systems assessment and is developed by health services supported by STRIVE coordinators to guide strategies for the health services over the next year of the trial and aimed at improving sexual health service delivery. The strategies may involve better application of existing procedures. For example discussion of STI best practice at weekly service meetings and regular data review, or new innovations such as STI testing visual reminder cards placed in clinics; implementing systematic approaches for collection of urine specimens and ensuring electronic recalls are installed within patient information management systems. The action plan is reviewed at three months and updated six monthly following the site visits.

#### Indicator reports

Health service staff and managers are provided with six monthly reports based on the best practice indicators, to discuss at the feedback meetings. These quantitative reports show the extent of improvements towards the targets. In addition to the best practice indicators, the reports also provide information on the proportion of community residents in the target age group who attended the health service during the period. Population denominators are derived from the number of Aboriginal residents who attended the health service in the preceding two years.

#### Clinical performance payments

After randomisation to the SHQIP, health services receive incentive payments on a six monthly basis, except for testing coverage, which is assessed annually. Payments are calculated on the basis of progress towards best practice targets. Services receive payments of $100 (AUD) for each episode of treating patients with STI symptoms at consultation; for treating a person diagnosed with an STI within seven days of the laboratory result being received at the clinic; and for re-testing each individual diagnosed with an STI at three months. Services also receive $100 per contact tested and treated within two weeks of being named. For a 10% or more improvement in any indicator in the subsequent period, the service receives a $500 payment. Services which reach the targets receive an additional $500, once only.

### Data collection methods and variables

The trial uses a number of methods to collect data (Table 2).

**Table 2:** Tools, methods and description of information collected in the trial

**Tool**	**Method and frequency**	**Description of data collected**
Site description assessment	• Conducted by regional STRIVE Coordinators at baseline and the start of each year in all health services	*Broad description of:*
	• Updated annually with any contextual information that may impact on delivery of STI services	(i) Current sexual health activities, including health promotion activities
Systems Assessment	• Conducted by regional STRIVE Coordinators annually in health services randomised to the SHQIP	*Information on:*
		(i) Health hardware
		(ii) Clinical services
		(iii) Patient information systems
		(iv) Health promotion
		(v) Organizational commitment to sexual health
		(vi) Surveillance and evaluation
Quantitative STI testing and clinical management data	• Collated from one or more of the following sources:	(i) Patient consultation data and associated demographics
	(i) Health service patient information management systems	(ii) STI testing, retesting and treatment outcomes Laboratory test result
	(ii ) STI templates within patient management systems	
	(iii) Contact tracing forms	
	(iv) Laboratory data	
	• Extracted 6 monthly for all health services	
STI prevalence assessment	• Each person attending the clinic aged 16-34 will be offered a STI screening test for all three STIs	(i) Prevalence of chlamydia, gonorrhoea and trichomonas in 16-34 year olds in STRIVE trial clusters
	• 50 men and 50 women from each cluster will be included	
	• Takes place on an annual basis in all health services	

### Prevalence assessment

At the start of each year and prior to initial randomisation the prevalence of chlamydia gonorrhoea and trichomonas will be assessed in clients attending participating health services. The assessment will involve screening 50 male and 50 female resident clients aged 16–34 years per cluster. All those in the age group attending the health service in the prevalence assessment period will be offered testing, unless they have had a test within the preceding three months. For clusters with more than one primary health care centre, the target numbers of 50 men and women will be allocated proportionately according to the number of health services and the size of the community served. The prevalence will be assessed again at the end of the study (Figure 1).

### Outcome measures

Primary outcomes are:

1. Prevalence of chlamydia, gonorrhoea or trichomonas in male and female Aboriginal residents aged 16–34 years, as measured using the trial prevalence assessment (Table 2); and

2. Performance in clinical service activity based on the best practice indicators over the period of the trial (Table 1).

All outcomes will be calculated by cluster, service, gender, patient age group (16–19, 20–24, 25–29, 30–34 years), STI type, and the reason for attendance.

Secondary outcomes are:

1. Diagnoses of pelvic inflammatory disease (PID) and epididymitis

2. Feasibility and acceptability of research conducted in remote primary care settings.

### Sample size

Power calculations were performed using the method of Hayes and Bennet [34] for cluster randomised trials. Estimates of between and within cluster variability in the primary end point were based on prevalence of the selected STIs in 2006 (the most recent prevalence data available) among Aboriginal people aged 14–34 years in central Australia [35]. Prevalence of chlamydia and gonorrhoea was estimated to be 15%, with an average 65 people tested in each cluster for chlamydia and gonorrhoea. Based on the 2006 notification data, between community variability was estimated as corresponding to a standard deviation of 8.45%, and the within community co-efficient of variation of 0.42. A total of 14 clusters are needed to detect a reduction in chlamydia and gonorrhea prevalence from 15% in control clusters to 7.5% in SHQIP clusters with 80% power and allowing for differing average numbers of people screened in each cluster (2-alpha = 0.05, with no adjustments for multiple comparisons), and equal numbers of clusters in each arm.

### Statistical analysis plan

Primary analysis will compare randomised clusters using an intention to treat approach. Characteristics of clusters will be summarized at baseline and across study arms (SHQIP or usual practice).

Analyses of the primary endpoint will be based on generalized linear mixed models to account for within and between cluster variability. The generalized estimating equation approach, with robust standard errors, will be adopted, using STATA 12.0 (College Station, TX, USA) statistical analysis software. Initial analyses will be simple, unadjusted comparisons of randomised clusters. If there appear to be any important imbalances between randomised groups in terms of baseline covariates, adjusted analyses will also be performed. All effects will be estimated with a 95% confidence intervals and p-values from the corresponding hypothesis tests. Statistical significance will be taken as two sided p-value less than 0.05, with no adjustment for multiple comparisons.

There will be two main analyses. First, after one year of follow-up there will be a comparison between the first and second round communities, assessing the effect of the intervention after one year. Second, after two years follow-up there will be a comparison between the first round and third round clusters, assessing the effect of the intervention after two years. The investigators recognize that a range of sexual health program activities will be taking place across communities throughout the duration of STRIVE. Systematic monitoring of these activities as described in Table 2 will allow us to compare them, both qualitatively and quantitatively, between SHQIP and usual practice communities.

There are a few envisaged limitations to the STRIVE protocol. Firstly the STRIVE study will determine if implementing a CQI approach is effective in reducing STI rates, but will not address the issue of testing for STIs among community members who do not attend a health service. At this point we do not know what proportion of the community do not attend a health service, especially in the younger age groups within the 16–34 year age bracket.

Secondly the STRIVE study is powered to detect a 50% decline in STI prevalence but it is possible that reductions may be less than this. However, a 50% reduction in prevalence would still leave rates at between 4 and 30 times higher, and the level of STI care provided after the SHQIP program will still be comparable to what is provided in health services accessible to Australians not living in remote communities.

Other key challenges to STRIVE are those characteristic of remote environments and include the need to sustain the motivation of clinic staff to participate in STRIVE while addressing the multiple competing health priorities, as well as high staff turnover.

## Discussion

Rates of STIs in remote Aboriginal communities in Australia are unacceptably high and STRIVE will support health services to achieve ‘best practice’ STI care by evaluating the efficacy of a SHQIP in decreasing STI prevalence. A strength of STRIVE is its ethical and robust design and large sample size of 68 remote communities. To our knowledge, STRIVE is the largest randomised controlled trial ever conducted among Aboriginal and/or Torres Strait Islander adults in Australia. The trial outcomes will enhance understanding of STI control and provide evidence of the effectiveness of SHQIPs in endemic communities as program data do not provide sufficient evidence of the effectiveness of programs.

Results will provide valuable and much needed information to guide STI clinical practice and programs in remote Aboriginal settings and other vulnerable communities with endemic rates of STIs.

## Competing interests

All authors declare no competing interests.

## Authors’ contributions

JW and SMcG prepared the manuscript. All other authors reviewed the manuscript and contributed to the research protocol. All authors read and approved the final manuscript.

## Pre-publication history

The pre-publication history for this paper can be accessed here:

http://www.biomedcentral.com/1471-2334/13/425/prepub
